# Validation of the portable virtual reality training system for robotic surgery (PoLaRS): a randomized controlled trial

**DOI:** 10.1007/s00464-021-08906-z

**Published:** 2021-12-06

**Authors:** Sem F. Hardon, Anton Kooijmans, Roel Horeman, Maarten van der Elst, Alexander L. A. Bloemendaal, Tim Horeman

**Affiliations:** 1 Department of Surgery, Amsterdam UMC–VU University Medical Center, Amsterdam, The Netherlands; 2 Department of Biomechanical Engineering, Delft University of Technology, Mekelweg 2, 2628 CD Delft, The Netherlands; 3 Department of Surgery, Reinier de Graaf Gasthuis, Delft, The Netherlands; 4 Faculty of Mechanical, Maritime and Materials Engineering (3mE), Delft University of Technology, Delft, The Netherlands

**Keywords:** Robot surgery, Simulation training, Learning curve, Patient safety-LMIC

## Abstract

**Background:**

As global use of surgical robotic systems is steadily increasing, surgical simulation can be an excellent way for robotic surgeons to acquire and retain their skills in a safe environment. To address the need for training in less wealthy parts of the world, an affordable surgical robot simulator (PoLaRS) was designed.

**Methods:**

The aim of this pilot study is to compare learning curve data of the PoLaRS prototype with those of Intuitive Surgical’s da Vinci Skills Simulator (dVSS) and to establish face- and construct validity. Medical students were divided into two groups; the test group (*n* = 18) performing tasks on PoLaRS and dVSS, and the control group (*n* = 20) only performing tasks on the dVSS. The performance parameters were Time, Path length, and the number of collisions. Afterwards, the test group participants filled in a questionnaire regarding both systems.

**Results:**

A total of 528 trials executed by 38 participants were measured and included for analyses. The test group significantly improved in Time, Path Length and Collisions during the PoLaRS test phase (*P* ≤ 0.028). No differences was found between the test group and the control group in the dVSS performances during the post-test phase. Learning curves showed similar shapes between both systems, and between both groups. Participants recognized the potential benefits of simulation training on the PoLaRS system.

**Conclusions:**

Robotic surgical skills improved during training with PoLaRS. This shows the potential of PoLaRS to become an affordable alternative to current surgical robot simulators. Validation with similar tasks and different expert levels is needed before implementing the training system into robotic training curricula.

**Supplementary Information:**

The online version contains supplementary material available at 10.1007/s00464-021-08906-z.

The utilization of robotic systems for common surgical procedures is rapidly increasing [1–4]. These systems allow the surgeon to operate with enhanced accuracy and precision with respect to instrument handling skills. Robotic surgery, a completely different approach in many aspects compared to more conventional surgical approaches, is seen as a progression by many. However, the way the instruments are operated, combined with the lack of tactile feedback requires a learning curve to overcome, even for experienced open and laparoscopic surgeons. To aid in this need, a dedicated training curriculum needs to be implemented before commencing robot-assisted surgery in the operating room (OR).

Simulation training allows surgical trainees to train in a low-stakes environment that enables deliberate practice [5], without compromising patient safety [6]. However, because of the high cost of current commercially available stand-alone simulators [7, 8], many hospitals rely on using their surgery robot’s downtime for training, after regular work hours. Ideally, to optimize the efficient use of the robot in the OR for real patients and to facilitate an adequate amount of training hours to overcome the robotic surgery learning curve, novice robotic surgeons should be able to practice their robotic skills on a more flexible basis. In less developed countries, hospitals often have fewer financial means to purchase dedicated training systems for their surgical robots. Here, robotic surgeons often need to travel to European or North American training centers to rent time on a robotic simulator. Upon returning to their home country, they are offered very little opportunity to retain these skills. Therefore, it is expected that local simulator training would drastically improve acquisition and retention of skills in a more affordable and sustainable way.

To cater for the demand for affordable and efficient ways to train robotic surgeons, a Virtual Reality (VR) training system called ‘Portable Laparoscopic Robotic Surgery’ (PoLaRS) was developed in collaboration with the Delft University of Technology (Delft, The Netherlands). It is designed to train fundamental robotic skills on a remote basis at an affordable cost of less than 25 k Euro per system. The technical skills that are acquired on the PoLaRS system could potentially be transferred to any currently available surgical robotic system to continue the learning curve and to hone these skills. Therefore, the availability of affordable simulators can be helpful in supporting localized training opportunities, thereby greatly improving the acquisition and retention of skills. This study aims to compare performance parameters of the PoLaRS system and the da Vinci Skills Simulator (dVSS) by establishing learning curves, as well as to assess validity evidence. It is hypothesized that construct validity can be indicated by similarity in learning curves of both systems, and prior training on the PoLaRS device will result in a shorter learning curve compared to the da Vinci system.

## Materials and methods

### Study design and participants

Medical interns at the Leiden University Medical Center (Leiden, the Netherlands) were recruited for voluntary participation in this initial validation study. Participant demographics and baseline characteristics (Supplemental File 1, 2) were obtained by a pre-course survey [9]. All participants first completed a pre-test the ForceSense box trainer, to establish a baseline level of technical skills for laparoscopy, such as hand–eye coordination, depth perception, and bimanual dexterity. Participants completed two tasks, with three trials per task as a baseline assessment (Table 2). After completion of the pre-test, participants were randomized into two groups (“test” group and “control” group) with https://www.randomizer.org/, using block randomization and a block size of four. Thereafter, participants of the test group completed two exercises on the PoLaRS prototype, performing each exercise three times (see Supplemental File 3). The control group participants viewed an instructional video from the company giving an overview of the da Vinci system instead. Afterwards, both groups performed two exercises on the da Vinci system, both performing each exercise three times. A questionnaire was sent to test group participants asking for feedback regarding face and construct validity of the PoLaRS and da Vinci systems. Due to the nature of this study and only passively controlled interface movements of both training systems, IRB approval and written consent are not required (Supplemental file 4).

### Hardware and systems

In this study, the following equipment was used:

1. Laparoscopic box trainer with an endoscopic camera, Maryland graspers and ForceSense measuring system;
2. PoLaRS prototype;
3. da Vinci console with Skills Simulator add-on.

The ForceSense laparoscopic system (MediShield B.V., Delft, the Netherlands) consists of two TrEndo instrument tracking sensors, one ForceTRAP tissue interaction force sensor, an endoscopic video camera, and a Windows tablet with pre-installed ForceSense.NET software (Medishield B.V., Delft, the Netherlands). These items were installed in a T5 boxtrainer (3-Dmed, Franklin OH, USA). Two endoscopic Maryland graspers (Applied Medical Corp, Rancho Santa Margarita CA, USA) were inserted into the TrEndo instrument tracking sensors (Supplemental File 5).

**Fig. 1:**
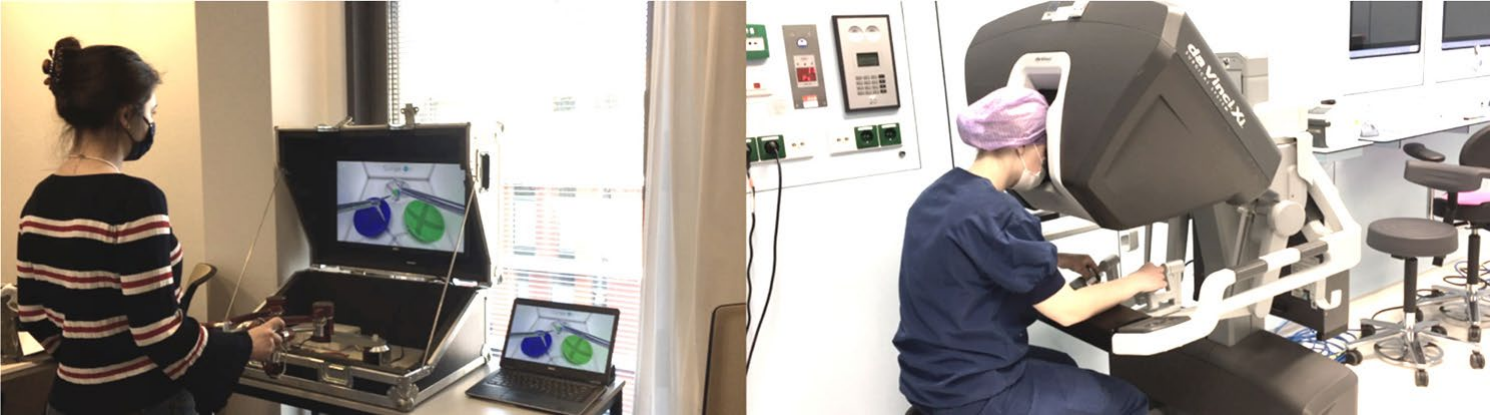
Overview of PoLaRS prototype (left) and dVSS system (right) in use

The Portable Laparoscopic Robotic Surgery system (PoLaRS) (Fig. 1) is a prototype system consisting of a console with two handles, each with seven degrees of movement and a custom-built I/O circuit board to translate the movements of all parts of the arms into electrical signals. The PoLaRS virtual reality (VR) software, programmed in the game engine Unity (Unity Software, Inc., San Francisco CA, USA) [10], was run on a regular Windows laptop (see Supplementary Fig. 3). The complete system can be installed on a table top surface at any height and can therefore be used from both sitting (similar to the dVSS) and standing position.

The dVSS (Intuitive Surgical, Inc., Sunnyvale CA, USA) is a VR system that is added to the console of a da Vinci Xi surgical robot (Fig. 1 Right). It offers a wide variety of exercises, ranging from instrument handling exercises to complete simulations of surgical procedures.

### Software and data collection

The ForceSense system makes use of pre-installed ForceSense.NET software, which records the real-time video feed from the endoscopic video camera and the force parameters and raw data collected by the ForceTRAP sensor. All data collected during the performance of a task are stored on the ForceSense.NET server, which is accessible with a personal user account.

In trainer mode, the PoLaRS makes use of a VR environment in which several exercises can be performed, similar to the da Vinci Skills Simulator. On request, visual and tactile force feedback is provided in case of instrument-environment collisions. In the PoLaRS software, when a collision is detected, a 3-s ‘cool-down’ period is applied, after which another collision can be detected. This ‘cool-down’ period is effective in a 10 mm radius around the center of the active collision. This means that longer collisions and/or larger movements cause multiple collision counts compared to a single hit as registered by the dVSS. From all parameters, the time to completion (s), left and right instrument path length (mm) and the number of collisions between instruments and non-movable environmental objects were used in this study. All performance metrics are saved locally in a designated folder according to the date and time of the trial.

The dVSS software provides training exercises for handling the da Vinci Xi system controls. The software includes training statistics, and every trial is scored according to technical performance. Exercises available on the dVSS system can be made by either Intuitive Surgical itself, or by Mimic Simulation (Mimic Technologies Inc., Seattle, WA USA). Both provided a performance score after each trial. Time to completion (s), Path length for both left and right instruments (e.g., Economy of motion) (cm), and number of instrument collisions were recorded. Intuitive Surgical records a single Instrument collisions metric, while Mimic-developed exercises record different types of collisions separately, namely Instrument-Endoscope collisions, Endoscope-Environment collisions, and Instrument-Instrument collisions. To provide comparability between them, Mimic’s collision metrics were summarized into a single Collisions parameter. All scoresheets are saved consecutively and locally and can be accessed by viewing the exercise history.

### Main study parameters

The main performance parameters that were used in this study were: time taken to complete the task; instrument path length left and right; and the number of collisions (Table 1). There were no limits to the instrument path length or the number of collisions. However, a time limit of 300 s per task was set for logistical considerations. Trials exceeding the time limit were marked as “did not finish” and were excluded from the analysis. The ForceSense system recorded previously validated force-based parameters for the objective assessment of tissue handling skills [11–14].

**Table 1:** Performance parameters

Parameter	Unit	Description
Time	Seconds	Measured from beginning of exercise to the completion of the exercise
Path length	Millimetres (PoLaRS) Centimetres (dVSS)	Total distance traveled by the tips of the left and right instruments during the task. Lower path length indicates more efficient movements
Number of collisions	#	The number of times an instrument or object collides with a solid surface (e.g., wall) during an exercise
Force penalties *(ForceSense)*	#	The number of times a force above N is applied to the task board
Max force *(ForceSense)*	Newtons	The maximum amount of force applied to the task board

### Study protocol

**Fig. 2:**
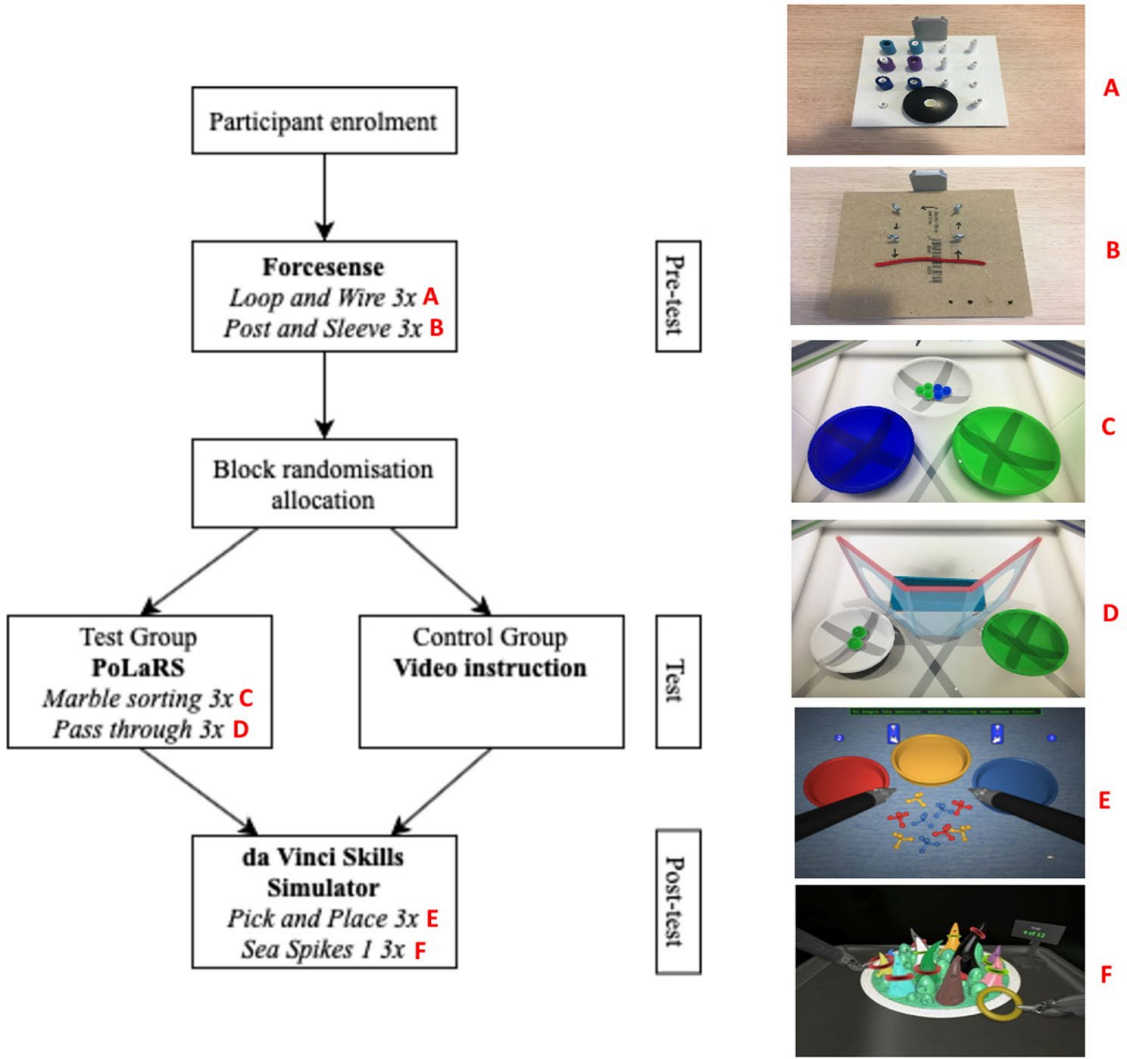
Flowchart of study design

Participants first completed a pre-test on the ForceSense laparoscopic system (Fig. 2). The pre-test consisted of two previously validated fundamental laparoscopic skill (FLS) tasks [13–15]. Participants completed three trials per task as a baseline assessment. The first task, ‘Loop and Wire’, required participants to guide a piece of pipe cleaner (metal wire with brushes) through several loops in order. The second task, ‘Post and Sleeve’, presented a board with posts, and six rubber sleeves placed on the left half of these posts. The participants were required to reposition the sleeves to the right half of the board. Furthermore, the sleeves were to be picked up with the left hand, transferred to the right hand in mid-air, and positioned on one of the correct posts with the right hand. Supplemental Files 6 and 7 provides more detailed descriptions and images of these tasks. After completion of the pre-test, participants were randomized into two groups (test group and control group) with https://www.randomizer.org/, using block randomization with a block size of four. After randomization, the test group participants were instructed to complete two exercises on the PoLaRS system. In the first exercise (Supplemental File 8), participants were tasked with sorting marbles according to their color. They were instructed to sort the green marbles with their left hand and the blue marbles with their right hand to ensure bimanual performance. The second task (Supplemental File 9) consisted of picking up marbles and feeding them through circular cut-outs in walls, making use of depth perception. Three trials of each exercise were performed. The PoLaRS system was set up the skills lab Reinier de Graaf Gasthuis teaching hospital, which was not accessible to the control group participants. Instead, they were asked to view a short introductory video on the da Vinci system, showing the different elements of the system, but not showing how the controls relate to the movement of the instruments. A link to this video can be found in Supplemental File 3.

The post-test was performed on a dVSS unit, connected to the da Vinci Xi console (Intuitive Surgical, Sunnyvale CA, USA) in the OR at the Reinier de Graaf Gasthuis teaching hospital. All participants completed two exercises, with three trials each. Contrary to the PoLaRS system, the dVSS has many different exercises to choose from. Thus, exercises were selected beforehand by AK and BB to reflect the PoLaRS exercises, which are similar in nature, without the use of foot pedals, clutch, or camera handling. ‘Pick & Place’ and ‘Sea Spikes 1’ were selected for this study, as can be seen in Supplemental Files 10 and 11.

### Face and construct validity

Participants in the test group were asked to fill out a questionnaire (Supplemental File 12) in which they could provide feedback on the PoLaRS and da Vinci systems after completion of all test sessions. This questionnaire contained both open and closed questions. Closed questions regarded face- and content validity and were answered using a 1–10 Likert scale. Open questions regarded the benefits and possible points of improvement for both the PoLaRS prototype and the DaVinci system. Responses were grouped into categories and interpreted by AK.

### Statistical analysis

The data from all systems were collected and analyzed using IBM SPSS (version 27.0, SPSS, Inc., Chicago IL, USA). Differences were considered significant at *p* < 0.05. Baseline performance was compared using the third trial of each exercise on the ForceSense system. Normality tests using Shapiro–Wilks tests were performed for all collected parameters. For normal distribution, unpaired t-tests were used to compare the data. In the case of non-normal distribution, the Mann–Whitney U test was performed. Parameter data were compared in each group between the first and last trials of the test and post-test phases to identify learning effects, and between the two groups in the post-test phase to identify the effects of prior PoLaRS training on performance. Questionnaire data were used to indicate whether face and construct validity were reached based on an average score above six on a 1–10 Likert scale.

## Results

A total of 528 trials executed by 38 enrolled participants were measured and included for analyses. All participants completed the pre-test, after which 18 were randomly placed in the test group, and 20 in the control group. Both groups had similar baseline characteristics (Supplemental File 2), and both groups performed similarly on the ForceSense system (Table 2). In the test group, 16 participants completed the test and post-test sessions, while in the control group 18 participants completed all the sessions. During the pre-test, two participants exceeded the 5-min time limit on a single task and were therefore excluded from further analysis. Due to technical difficulties, two participants could not complete all tasks on the PoLaRS system, and one participant could not perform the ‘Sea Spikes’ exercise on the dVSS. One participant exceeded the 5-min time limit on one PoLaRS task, and one participant exceeded the 5-min time limit on one dVSS task trial.

**Table 2:** Baseline performance

	ForceSense ‘Loop and Wire’, trial 3		
	Control (*n* = 19)	PoLaRS (*n* = 17)	Sig.*
Time (s)	83.28 (50.80–164.92)	95.38 (53.20–239.30)	.232
Path length (mm)	3310.47 (1523.74–8374.70)	3513.24 (1983.41–11,894.30)	.731
Force penalties	3 (0–42)	3 (0–53)	.975
Max force (N)	2.59 (1.31–5.23)	2.54 (1.24–6.86)	.661

	ForceSense ‘Post and Sleeve’, trial 3		
	Control (*n* = 19)	PoLaRS (*n* = 17)	Sig.*
Time (s)	96.26 (61.98–157.70)	107.20 (63.70–150.60)	.379
Path length (mm)	3557.36 (2473.29–7929.36)	3879.78 (2569.44–7805.82)	.900
Force penalties	1 (0–51)	1 (0–45)	.802
Max force (N)	2.35 (1.60–4.46)	2.37 (1.30–7.60)	.950

Median (range), *Mann–Whitney U

### Learning curve PoLaRS and da Vinci skills simulator

**Fig. 3:**
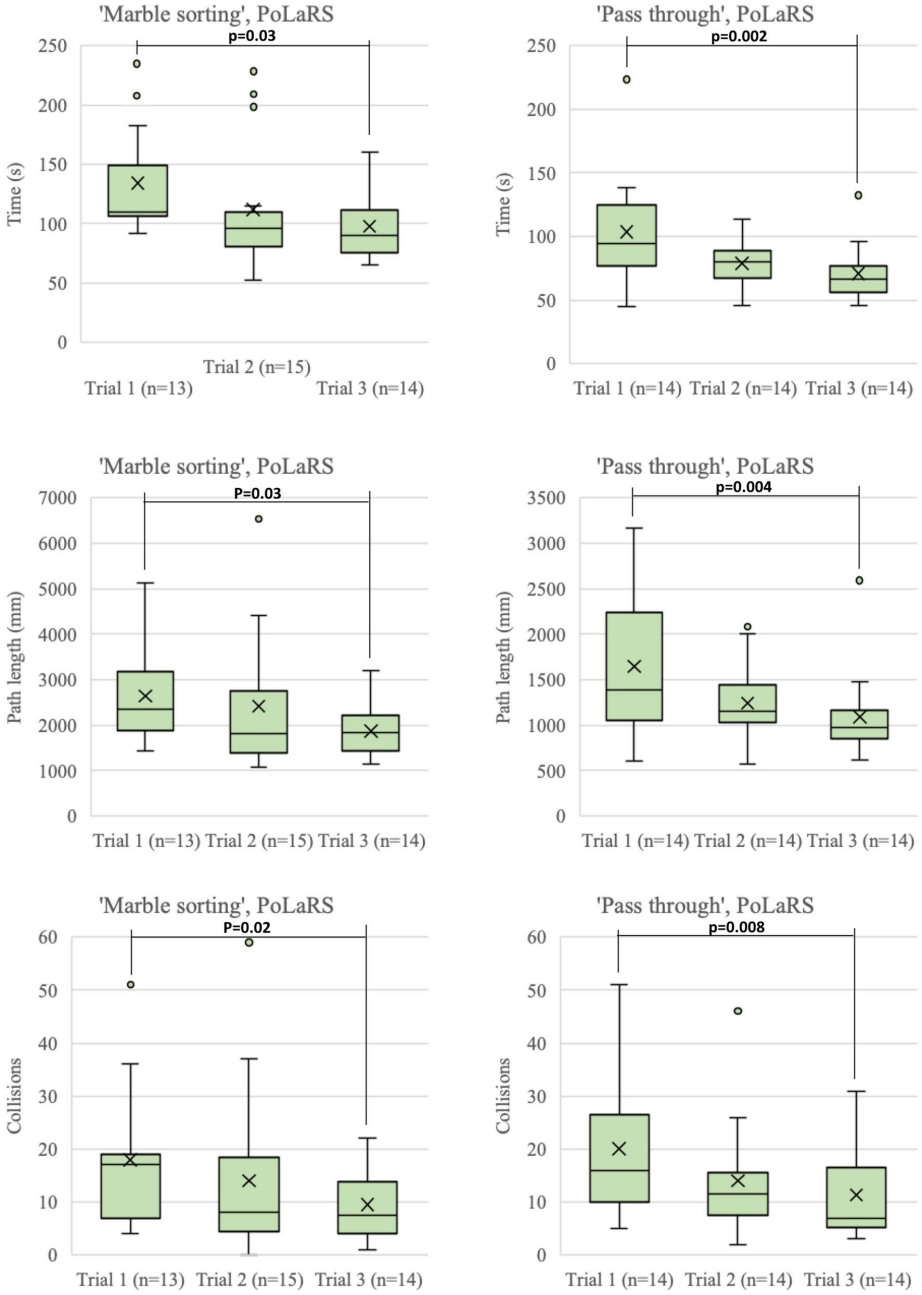
Boxplots of performance parameters of both ‘Marble sorting’ and ‘Pass through’ tasks on PoLaRS. ‘X’ indicates mean. Significant differences as indicated by the Wilcoxon Signed Ranks test are indicated with a *p*-value < 0.05

**Fig. 4:**
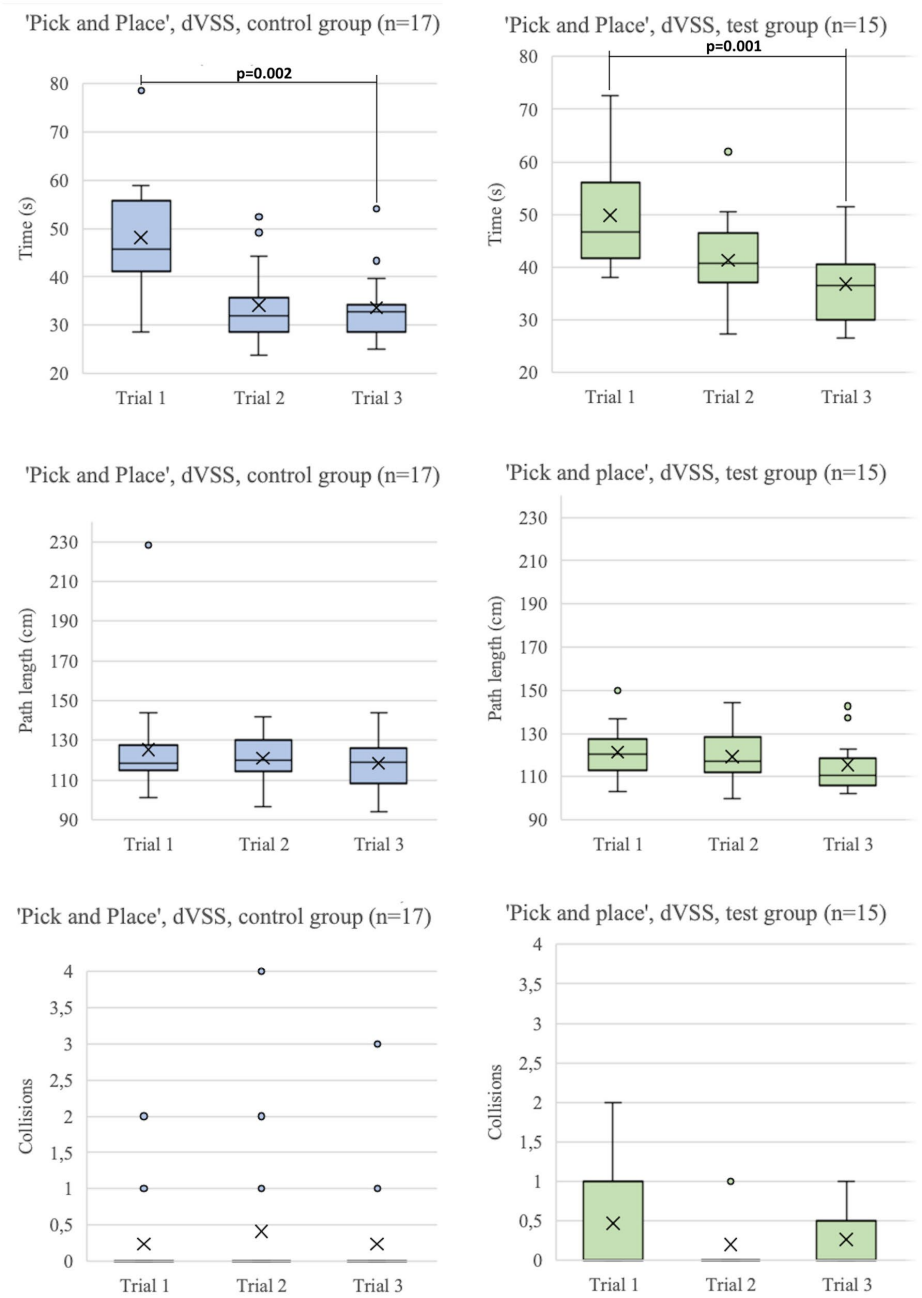
Boxplots of performance parameters of ‘Pick and Place’ task on dVSS. ‘X’ indicates mean. Significant differences as indicated by the Wilcoxon Signed Ranks test are indicated with a *p*-value < 0.05

**Fig. 5:**
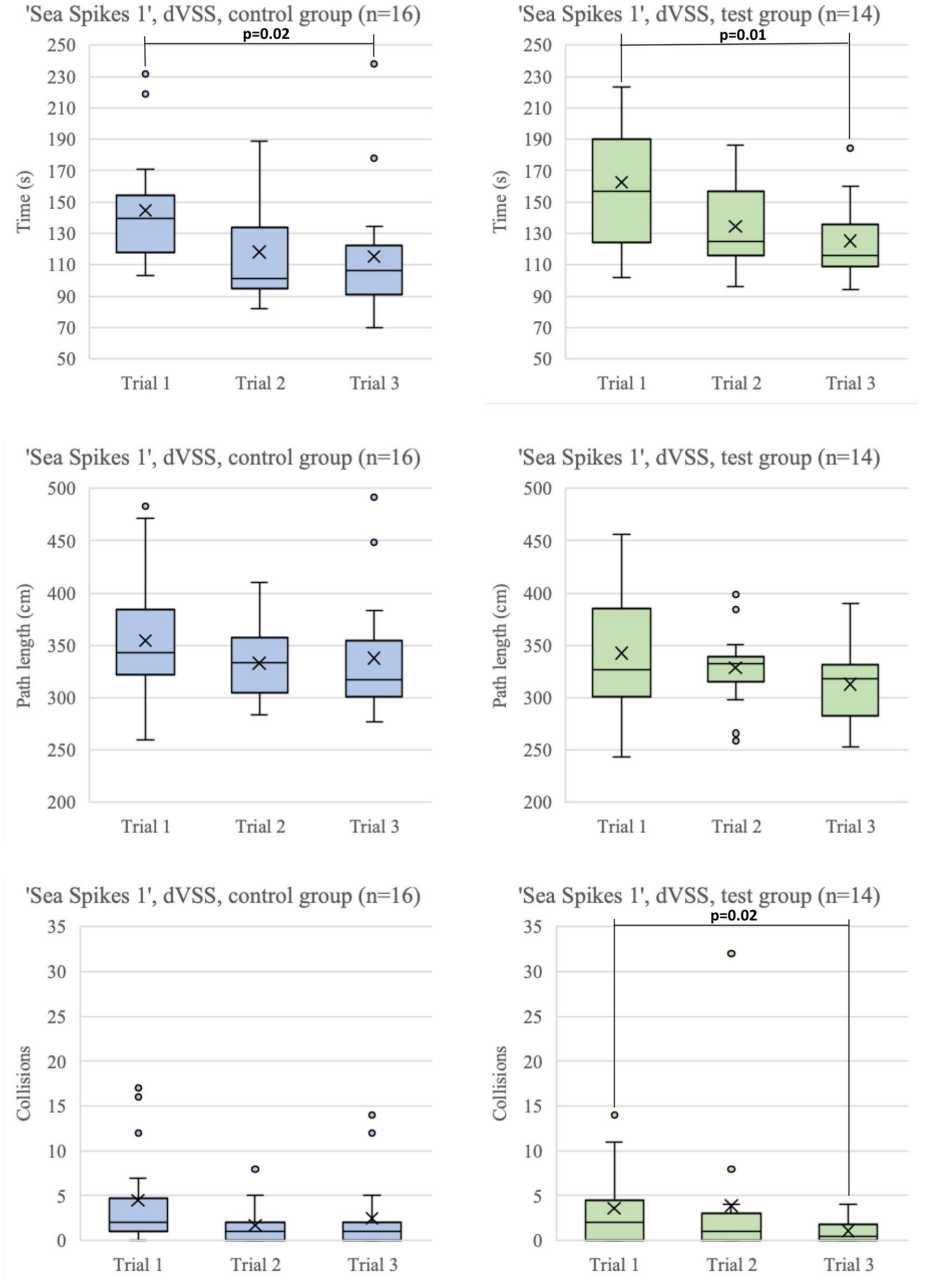
Boxplots of performance parameters of ‘Sea Spikes 1’ task on dVSS. ‘X’ indicates mean. Significant differences as indicated by the Wilcoxon Signed Ranks test are indicated with a *p*-value < 0.05

Figure 3 shows boxplots of the Time to completion, Path length and Collisions of all three trials performed on the ‘Marble sorting’ and ‘Pass through’ tasks of the PoLaRS system. Between the first and last trial of the ‘Marble sorting’ and ‘Pass through’ task, all parameters decreased significantly. Figure 4 shows boxplots of time to completion, path length and collisions of the three trials executed on the ‘Pick and Place’ task on the da Vinci system. Time to completion decreased significantly in both the control and test group between the first and last trial of each task. On the second trial, control group participants were significantly quicker to complete the task than the test group (*p* = 0.018). For the other parameters, no significant differences were found. Figure 5 shows boxplots of the trials performed on the ‘Sea Spikes 1’ task on the da Vinci system. A significant decrease in time to complete the task was found between the first and last trial for both the control and test group. Although not significant, the Path length showed a decreasing trend in both groups. Only for the test group the reduction of instrument collisions was significant.

### Questionnaire

**Fig. 6:**
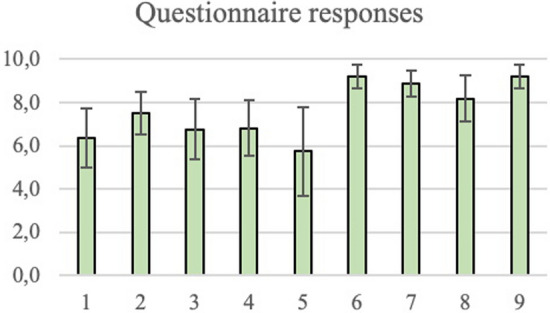
Bar diagram of questionnaire responses with standard deviation bars

The test group participants were sent a questionnaire after the testing sessions, asking for their feedback. Questions were either answered with a score of 1–10 on a Likert scale (Fig. 6) or a written answer. Analysis of the scores can be seen in Supplemental File 13, and written responses (in Dutch) can be found in Supplemental File 14.

The questionnaire also contained several open questions, where participants could provide feedback on the benefits and points of improvement of the PoLaRS prototype and the da Vinci system. According to the participants, the main benefit of the PoLaRS prototype was the fact that one can practice robotic surgery in a very low-stakes, accessible way (11 mentions). Further benefits were a large range of movement (two mentions), a good feeling of precision (one mention), and similarity of exercises to DaVinci (one mention). Points of improvement varied: suboptimal depth perception (five mentions), movement not as smooth as da Vinci (five mentions), lack of resistance in controllers (three mentions), ergonomics (three mentions), coordination feels counterintuitive (two mentions), less precision in small movements (one mention), controllers could be more like DaVinci (one mention), exercise realism/medically oriented exercises (one mention).

The benefits of the DaVinci system, according to participants, are the precision of movements (seven mentions), comfortable ergonomics (seven mentions), smooth movements (five mentions), ease of use (four mentions), three-dimensional view (three mentions), good resistance on controllers (one mention). Points of improvement mentioned for the DaVinci system include lack of tactile feedback (four mentions), controllers sometimes colliding with each other (two mentions), exercise realism/medically oriented exercises (two mentions), limited instrument reach (one mention), and suboptimal depth perception (one mention). 

## Discussion

In this study, we examined whether the PoLaRS prototype would provide a noticeable benefit in performance on the dVSS system. Although no significant differences were found, prior training on the PoLaRS prototype did not negatively impact performance on the da Vinci Skills Simulator and the questionnaire indicated that prior training with PoLaRS was helpful in performing on the dVSS system. Furthermore, performances in the test group seemed to have fewer outliers than the control group.

Although we expected some effect from training on the PoLaRS prototype prior to the dVSS, the individual learning curves reveal that it is most likely not the system but the training task that has a dominant influence on performance. In fact, for both systems, similar learning curve shapes and similar reductions in parameter data variation were observed for all tasks. Comparing the final trial on the first task and the first trial on the following task in the PoLaRS parameter data, large differences were found for each parameter. This same differences are found when switching from the first task to the second task on the dVSS. Moreover, when switching from PoLaRS to dVSS, a similar difference was found for all parameters without any disturbances. This indicates construct validity, as the data suggest that both systems are intuitive enough to be used as an effective extension of the human arm. To determine if the PoLaRS can be used as a training device for complex surgical tasks on a specific robotic platform, it is advised to validate similar training tasks for both systems with multiple experts [16]. In contrast to the da Vinci system, all subjects used the PoLaRS system in a standing position. As this could potentially influence the face and construct validation, future studies should be conducted with comparable body postures.

It is evident that the complexity of the training system and training tasks have an influence on the performance [11, 17]. In this study, we chose to test the participants with less complex position tasks and we did not instruct participants to use the finger clutch system on the da Vinci or PoLaRS Skills Simulators. However, in future studies, it is necessary to add a layer of complexity to the systems by adding decision-making and the use of clutch systems to investigate the influence on construct validity.

Although the questionnaire scores were lower for the PoLaRS system compared to the DaVinci Skills Simulator, an average score of 6.9 (SD 1.2) for the questions about the performance of PoLaRS indicates that face validity was established. A possible explanation for the lower scores is that the questionnaire was completed after all testing sessions were completed. Therefore, participants compared the PoLaRS system directly to the DaVinci system, rather than rate it on its own merit. In future studies it might be wise to have participants fill out these questions after each testing session. An interesting observation can be made from the questionnaire: when asked whether the exercises on the PoLaRS system made them perform better on the DaVinci system, participants varied greatly in their responses. Some participants did not feel like the PoLaRS system contributed to their performance on dVSS, while others did. Interestingly, there does not seem to be a correlation between those that performed well on the PoLaRS system and those that felt that the PoLaRS system contributed to their dVSS performance; the two highest raters (both rating 9/10) performed similarly to the two lowest raters (rating 2/10 and 3/10). This indicates that it may be difficult for participants to relate objective performance to factors of influence.

Concerning the questionnaire’s open questions, the three most important points of improvement were smoothness of movement, depth perception, and resistance in the controllers. However, the majority of them saw the benefits of using the PoLaRS system as an accessible and low-stakes means to develop their skills in operating surgical robots. Many participants noticed the lack of tactile feedback in both PoLaRS and dVSS systems, a feature of which implementation is currently being researched [18–20], which has been proven to reduce grip force during procedures [13].

### Technical difficulties

On two separate occasions, the dVSS computer experienced a freeze-up, where the participant could not finish their current task. It was believed to be an overheating issue; therefore, the computer was switched off completely and restarted after approximately five minutes. On one of the testing days, the computer froze two more times after restarting although the computer’s workload was markedly lower compared to other testing days where it functioned without issue. Although the influence of this kind of events on the learning curve during training seems minimal, it did raised the question whether a “freeze up” can happen during surgery and what the consequences can be.

### Future of surgical robotic systems

Many companies are working on surgical robots that are expected to enter the market in the coming years [21–23]. From what is shown to the public, it is expected that these systems may have novel ways of controlling robotic arms and instruments. Thus, it is possible that hospitals and outpatient surgical centers will own and operate different surgical robotic systems, with the possibility that surgeons need to work with several different systems within a short timeframe. It is important to acknowledge the challenges this may bring. For instance, what is the impact on patient safety when switching between robotic systems? Are, in this case, mandatory simulator hours a solution? Would surgeons need to be type-rated for specific systems, like pilots are for different types of aircraft? To anticipate these circumstances, it is vital that a robust training curriculum is in place, allowing robotic surgeons deliberate practice with these systems. Therefore, the PoLaRS system could become a system capable of adapting to these different controls, providing simulation training for a number of surgical robotic systems.

## Conclusion

Robotic surgical skills improved during training with PoLaRS. Although skills transfer between training tasks and systems was not demonstrated yet, this study shows the potential of PoLaRS to become an affordable alternative to current surgical robot simulators. Full construct validity needs to be established for different experience levels and training tasks before implementing this system into curricula.

## Supplementary Information

Below is the link to the electronic supplementary material.

Supplementary file1 (PNG 180 kb)

Supplementary file2 (PNG 242 kb)

Supplementary file3 (DOCX 14 kb)

Supplementary file4 (DOCX 18 kb)

Supplementary file5 (DOCX 13 kb)

Supplementary file6 (DOCX 15 kb)

Supplementary file7 (DOCX 14 kb)

Supplementary file8 (MP4 18086 kb)

Supplementary file9 (PDF 426 kb)

Supplementary file10 (PNG 448 kb)

Supplementary file11 (PNG 374 kb)

Supplementary file12 (PNG 396 kb)

Supplementary file13 (PNG 221 kb)

Supplementary file14 (PNG 211 kb)
