# Supplementary figures and images for: Validation of the portable virtual reality training system for robotic surgery (PoLaRS): a randomized controlled trial
Source: Surg Endosc. 2021 Dec 6;36(7):5282–92. doi: 10.1007/s00464-021-08906-z (PMC9160149; doi:10.1007/s00464-021-08906-z)

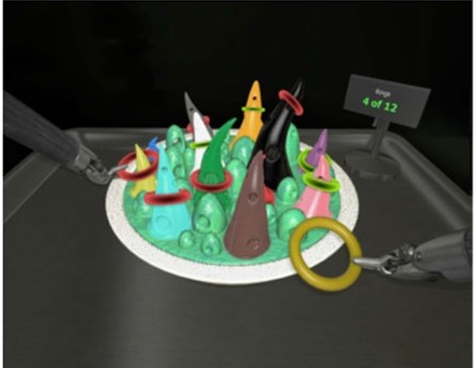

Supplement: Supplementary file 1 — Supplementary file1 (PNG 180 kb) [file 464_2021_8906_MOESM1_ESM.png]

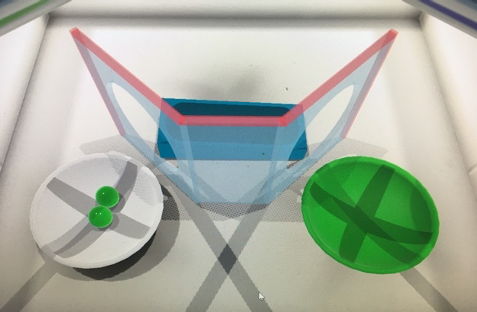

Supplement: Supplementary file 2 — Supplementary file2 (PNG 242 kb) [file 464_2021_8906_MOESM2_ESM.png]

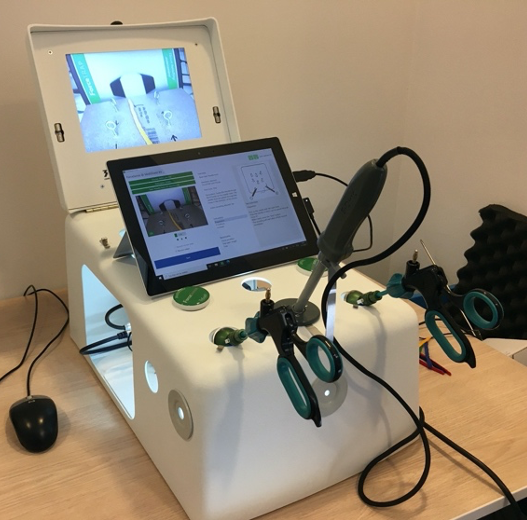

Supplement: Supplementary file 10 — Supplementary file10 (PNG 448 kb) [file 464_2021_8906_MOESM10_ESM.png]

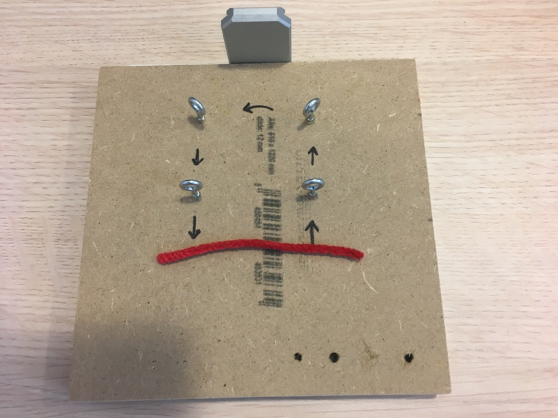

Supplement: Supplementary file 11 — Supplementary file11 (PNG 374 kb) [file 464_2021_8906_MOESM11_ESM.png]

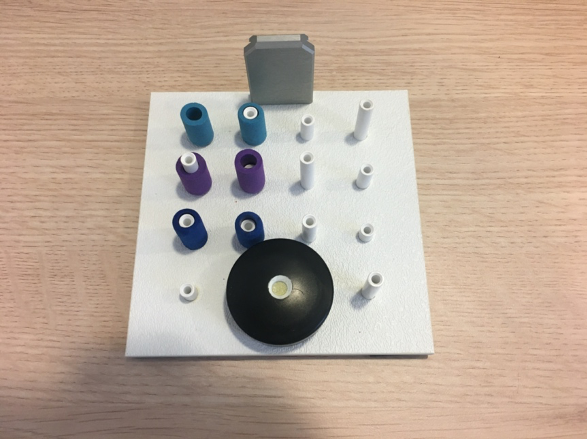

Supplement: Supplementary file 12 — Supplementary file12 (PNG 396 kb) [file 464_2021_8906_MOESM12_ESM.png]

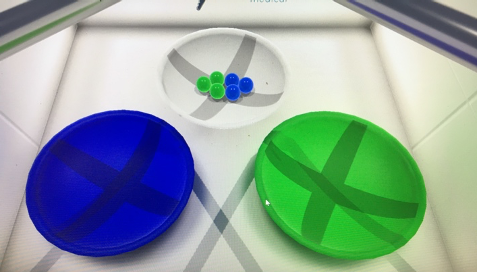

Supplement: Supplementary file 13 — Supplementary file13 (PNG 221 kb) [file 464_2021_8906_MOESM13_ESM.png]

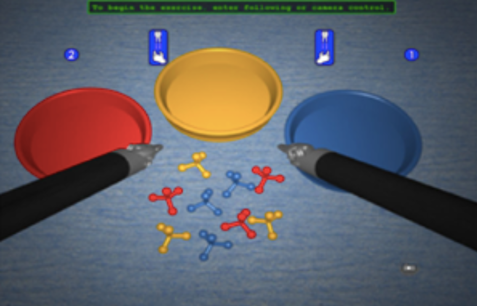

Supplement: Supplementary file 14 — Supplementary file14 (PNG 211 kb) [file 464_2021_8906_MOESM14_ESM.png]
