# Supplementary material for: Validation of the portable virtual reality training system for robotic surgery (PoLaRS): a randomized controlled trial
Source: Surg Endosc. 2021 Dec 6;36(7):5282–92. doi: 10.1007/s00464-021-08906-z (PMC9160149; doi:10.1007/s00464-021-08906-z)
Supplement: Supplementary file 3 — Supplementary file3 (DOCX 14 kb) [file 464_2021_8906_MOESM3_ESM.docx]

*Face and construct validity of PoLaRS and dVSS.*

| Face and construct validity of PoLaRS and dVSS. | |
| --- | --- |
| PoLaRS | |
| *Face validity* | Mean (SD) |
| Ergonomics and instrument handling | 6.3 (±1.38) |
| Realistic exercises | 6.8 (±1.39) |
| *Construct validity* | |
| Useful for hand-eye coordination training | 7.5 (±0.97) |
| Useful for training surgeons | 6.8 (±1.28) |
|  |  |
| Better performance on dVSS after training on PoLaRS | 5.8 (±2.05) |
| da Vinci Skills Simulator | |
| *Face validity* | Mean (SD) |
| Ergonomics and instrument handling | 9.2 (±0.54) |
| Realistic exercises | 8.2 (±1.05) |
| *Construct validity* | |
| Useful for hand-eye coordination training | 8.9 (±0.62) |
| Useful for training surgeons | 9.2 (±0.54) |
