# Supplementary material for: Validation of the portable virtual reality training system for robotic surgery (PoLaRS): a randomized controlled trial
Source: Surg Endosc. 2021 Dec 6;36(7):5282–92. doi: 10.1007/s00464-021-08906-z (PMC9160149; doi:10.1007/s00464-021-08906-z)
Supplement: Supplementary file 4 — Supplementary file4 (DOCX 18 kb) [file 464_2021_8906_MOESM4_ESM.docx]

*Questionnaire responses – Dutch.*

Participant #1

1. 8
2. 8
3. 7
4. 6
5. 5
6. 9
7. 9
8. 7
9. 9
10. Ik vond het prototype wel soepel bewegen en de range voor de bewegingen was wel goed.
11. Het grootste nadeel aan het prototype was het feit dat het scherm 2-dimensionaal is. Daardoor is het schatten van de diepte veel lastiger. Na een tijdje is het staan voor het prototype ook wat vervelend.
12. Het feit dat je diepte kan zien is een heel groot pluspunt. Verder zit je wel heel comfort op dat apparaat. De bewegingen zijn ook allemaal heel soepel.
13. Soms raakte ik per ongeluk de zijkant van het apparaat of raakten de twee controllers elkaar. Dat was nog wel eens een beetje vervelend. Ik zou niet goed weten hoe dat opgelost kan worden.

Participant #4

1. 7
2. 8
3. 6
4. 8
5. 7
6. 9
7. 9
8. 7
9. 9
10. De oefening kwam in enige mate overeen met de oefening op de DaVinci.
11. Ik had het gevoel dat de kalibratie niet helemaal goed was (kan ook aan mij liggen), en dat de reactiesnelheid niet zo snel was als die van de DaVinci.
12. Mate van precisie, soepelheid en vele mogelijkheden tot koppelen van instrumenten. Ergonomisch erg fijn.
13. Misschien meer 'realistische'/medische games?

Participant #5

1. 7
2. 7
3. 6
4. 6
5. 4
6. 9
7. 8
8. 8
9. 9
10. Goede manier om bekend te worden met de manier hoe robotchirurgie werkt zonder de logistiek van een OK complex.
11. Weerstand op de instrumenten zetten, bijvoorbeeld als de operateur de instrumenten niet aanraakt/geen kracht zet dat de instrumenten dan op dezelfde plek blijven staan net zoals bij de da Vinci robot.
12. Goede ondersteuning van de instrumenten ook als je als operateur geen kracht zet blijven de instrumenten op dezelfde plek.
13. Een keer probeerde ik iets over te pakken van het ene instrument naar de andere, maar dat lukte niet (weet alleen niet of dat aan mij lag).

Participant #6

1. 6
2. 6
3. 6
4. 7
5. 4
6. 9
7. 9
8. 9
9. 9
10. Zeker mooi en geavanceerd apparaat en geeft wel een duidelijk beeld van robotchirurgie weer
11. Ik vond persoonlijk de bewegingen in vergelijking met de Da Vinci wat stroever en ook de coördinatie had ik gewoon echt niet onder de knie, waardoor het inschatten van de beweging voor de oefeningetjes niet goed ging. Zou alleen echt niet weten hoe dit verbeterd kan worden.
12. Hele soepele, en natuurlijke bewegingen. Ook vond ik de coördinatie heel fijn aanvoelen.
13. Wat het apparaat misschien nóg beter zou maken is soort tactiele informatie teruggeven aan de vingers. Hoe dit in een klittenbandje verwerkt moet worden is een ander verhaal, maar als je nu iets oppakte zag je wel dat je het deed, maar niet echt het gevoel dat je het deed.

Participant #9

1. 5
2. 7
3. 7
4. 6
5. 7
6. 9
7. 9
8. 7
9. 10
10. Het is wel een voorbereidende manier voor de da Vinci robot en je went even aan de soort van besturing.
11. Voor m’n gevoel werkte het best contra-intuïtief en als ik dacht dat ik naar links zou gaan met m’n hand, ging ik bv naar rechts. Ik raakte er een beetje van in de war.
12. Werkt super intuïtief en gaat echt als vanzelf.
13. Soms botsten de twee ‘handen’ als terwijl dat op scherm niet te zien was, dus dat was wel gek.

Participant #11

1. 6
2. 8
3. 9
4. 7
5. 4
6. 10
7. 10
8. 9
9. 9
10. Hand oog coördinatie wordt goed getraind en er wordt geoefend met werken met elektronische chirurgie.
11. Ik vond dat PoLaRS lastiger was om te besturen omdat de dimensies minder goed zichtbaar waren, waardoor diepte lastiger was in te schatten dus daar kan zeker in worden verbeterd.
12. Doordat je echt met je ogen in zo’n kap zit, waren de dimensies helder en leek het net alsof je naar een 3D beeld aan het kijken was.
13. Wat mij betreft niet veel.

Participant #13

1. 7
2. 8
3. 9
4. 6
5. 7
6. 9
7. 9
8. 9
9. 9
10. Neemt geen tijd van de echte DaVinci in beslag. Geeft een idee wat je te wachten staat bij de da Vinci.
11. De besturing was een stuk “stroever” dan bij de da Vinci. Het reageerde ook minder op kleine bewegingen.
12. Makkelijk te besturen, heel sensitief voor kleine bewegingen.
13. Tactiele feedback die je wel bij laparoscopie bijv. hebt.

Participant #18

1. 4
2. 8
3. 7
4. 4
5. 6
6. 9
7. 9
8. 9
9. 10
10. Het geeft de mogelijkheid om zonder hoge druk/verwachtingen te oefenen voordat je met de da Vinci robot te werk gaat.
11. Het prototype komt qua beweeglijkheid en functies nog niet zo goed overeen. Het is lastig diepte te zien in het scherm/opdracht en lastig om de armen te bewegen.
12. Alles is tot in de puntjes uitgewerkt en werkt mee; van beweeglijkheid tot in de kleine details. Zeer gevoelig maar juist goed.
13. X

Participant #22

1. 7
2. 8
3. 7
4. 8
5. 9
6. 10
7. 9
8. 7
9. 10
10. Goede, laagdrempelige manier om te oefenen. Goed voor de basis.
11. Handvaten zoals bij de DaVinci robot maken.
12. Heel mooi apparaat, goed oefenprogramma en fijn om mee te werken.
13. Geen mening.

Participant #23

1. 7,5
2. 9
3. 7
4. 9
5. 9
6. 10
7. 9
8. 7
9. 9
10. Het geeft de mogelijkheid om op een andere manier te oefenen met robotchirurgie.
11. Het voelde af en toe een beetje tegennatuurlijk om te gebruiken. Diepte zien was moeilijker dan met de da vinci.
12. Makkelijk om diepte te zien en voelde natuurlijk aan
13. Dat het oefenprogramma wel werkt als het nodig is

Participant #25

1. 4
2. 5
3. 3
4. 6
5. 2
6. 9
7. 8
8. 10
9. 9
10. Het is een goede laagdrempelige manier om te beginnen met robotchirurgie, de simulaties zijn ook erg interessant en spreken goed aan op verschillende manieren van bewegen binnen de simulatie.
11. Naar mijn idee was de beweging die ik maakte met het apparaat niet altijd overeenkomend met wat ik op het scherm zag, hierdoor was de hand oog coordinatie die ik uitvoerde niet goed en ook best verwarrend.
12. Het feit dat je niets om je heen ziet behalve de simulatie zorgt ervoor dat je volledig opgaat in de simulatie, daarnaast zijn de bewegingen die je maakt heel verfijnd en komt het gevoel heel goed over.
13. Voor kleinere mensen zoals ik zelf was het soms lastig om in de simulatie bij het achterste deel te komen ivm armlengte, ik ga er vanuit dat dit in een chirurgische setting minder uit zal maken maar het maakte de simulatie iets lastiger voor mij.

Participant #27

1. 5
2. 7
3. 6
4. 7
5. 5
6. 8
7. 9
8. 9
9. 9
10. Het is een ander soort apparaat, en het geeft je wel een goed gevoel van secuurheid.
11. Ik vond hem wat houterig bewegen, al helemaal in vergelijking met de da Vinci, die was heel veel soepeler.
12. Echt een hele mooie techniek die elke kleine beweging goed opvangt. Erg secuur mee te werken.
13. Misschien meer informatie over hoe hard je ermee omgaat. Ik had geen idee bij het oefenen hoe hard ik iets vastpakte, en dat is natuurlijk wel belangrijk bij echte operaties.

Participant #29

1. 8
2. 8
3. 6
4. 9
5. 3
6. 9
7. 10
8. 8
9. 10
10. Toegankelijke (en leuke) manier om robotchirurgie te oefenen.
11. Bewegingen voelen (zeker vergeleken met de Davinci) houterig, de handgrepen zouden ook ergonomischer kunnen.
12. Prettige zithouding, bewegingen voelen natuurlijk en vloeiend aan.
13. -

Participant #32

1. 8
2. 8
3. 7
4. 7
5. 8
6. 9
7. 8
8. 7
9. 8
10. Werkt erg goed, handig om eerst met het prototype te oefenen en daarna de Davinci (denk ik)
11. Realistischere opdrachten/ omgeving die er iets meer uitziet als een menselijk lichaam
12. Werkt echt geweldig, hele nauwkeurige besturing, je zit echt “in” de opdracht/ operatie dat was heel fijn
13. Ook iets realistischere omgeving misschien?

Participant #35

1. 5
2. 7
3. 8
4. 7
5. 6
6. 9
7. 8
8. 9
9. 9
10. Het gaf een duidelijk oefening met handelen wat je te verwachten staat met de davinci robot.
11. Ergonomischer, ik ben vrij lang en kreeg beetje last van mijn rug. Ook is het diepte perspectief lastig in te schatten.
12. Ergonomisch en makkelijk te besturen.
13. Ook het diepte perspectief was vaak lastig in te schatten op de davinci robot

Participant #36

1. 7
2. 8
3. 7
4. 6
5. 6
6. 10
7. 9
8. 9
9. 9
10. Mogelijk om in veel verschillende vlakken te bewegen
11. Arm ondersteuning of iets meer weerstand, je schiet snel door.
12. Erg makkelijk te besturen en fijn ergonomisch model.
13. Misschien weerstand inbouwen zodat je kan voelen hoe hard je iets vastpakt
