# Supplementary material for: Validation of the portable virtual reality training system for robotic surgery (PoLaRS): a randomized controlled trial
Source: Surg Endosc. 2021 Dec 6;36(7):5282–92. doi: 10.1007/s00464-021-08906-z (PMC9160149; doi:10.1007/s00464-021-08906-z)
Supplement: Supplementary file 5 — Supplementary file5 (DOCX 13 kb) [file 464_2021_8906_MOESM5_ESM.docx]

*Questionnaire – English translation.*

Rate 1-10:

1. I found the PoLaRS prototype easy to control (ergonomics).
2. The tasks on the PoLaRS prototype are suitable for training hand-eye coordination.
3. The tasks on the PoLaRS prototype are realistic.
4. The PoLaRS prototype is suitable for training surgeons in robotic surgery.
5. By first performing the tasks on the PoLaRS prototype, I could perform the tasks on the da Vinci system better.
6. I found the da Vinci system easy to control (ergonomics).
7. The tasks on the da Vinci system are suitable for training hand-eye coordination.
8. The tasks on the da Vinci system are realistic.
9. The da Vinci system is suitable for training surgeons in robotic surgery.

Answer:

1. What is good about the PoLaRS prototype?
2. What can be improved on the PoLaRS prototype?
3. What is good about the da Vinci system?
4. What can be improved on the da Vinci system?
