# Supplementary material for: Validation of the portable virtual reality training system for robotic surgery (PoLaRS): a randomized controlled trial
Source: Surg Endosc. 2021 Dec 6;36(7):5282–92. doi: 10.1007/s00464-021-08906-z (PMC9160149; doi:10.1007/s00464-021-08906-z)
Supplement: Supplementary file 6 — Supplementary file6 (DOCX 15 kb) [file 464_2021_8906_MOESM6_ESM.docx]

*Participant registration form – English translation.*

| Name* |  |
| --- | --- |
| Age |  |
| Sex | Male / Female |
| Dominant hand | Left / Right |
| Current educational level | Medicine Bachelor’s year 1 / 2 / 3  Medicine Master’s year 1 / 2 / 3  Other: |
| Do you have prior experience handling laparoscopic and/or surgical robotic equipment? | Yes / No  In case of Yes, estimated time in minutes: |
| On average, how many hours per week do you play videogames? |  |

*All collected data will be processed anonymously.

I hereby agree to the collection of anonymous data during this workshop for use in scientific research.

I hereby agree to the recording of video footage of performances on the training systems used during this workshop.

Date: …………………………………

Signature: ………………………………
