# Supplementary material for: Validation of the portable virtual reality training system for robotic surgery (PoLaRS): a randomized controlled trial
Source: Surg Endosc. 2021 Dec 6;36(7):5282–92. doi: 10.1007/s00464-021-08906-z (PMC9160149; doi:10.1007/s00464-021-08906-z)
Supplement: Supplementary file 7 — Supplementary file7 (DOCX 14 kb) [file 464_2021_8906_MOESM7_ESM.docx]

*Baseline characteristics.*

| Baseline characteristics | | | |
| --- | --- | --- | --- |
|  | Control (n=20) | PoLaRS (n=18) | Sig. |
| Age (yrs) | 22 (20-27) | 21 (20-24) | .076 |
| Sex* (% female) | 60.00 | 61.11 | .604 |
| Right-handed* (%) | 75.00 | 94.44 | .184 |
| Medicine year; Other** | 4 (2-6); n=2 | 3 (1-6); n=0 | .085 |
| Experience in laparoscopy (min) | 0 (0-30) | 0 (0-60) | .331 |
| Gaming habits (h/week) | 0 (0-5) | 0 (0-14) | .501 |

Median (range), Mann-Whitney U. *Fisher’s Exact test. **Medicine pre-master program.
