# Supplementary material for: Validation of the portable virtual reality training system for robotic surgery (PoLaRS): a randomized controlled trial
Source: Surg Endosc. 2021 Dec 6;36(7):5282–92. doi: 10.1007/s00464-021-08906-z (PMC9160149; doi:10.1007/s00464-021-08906-z)
Supplement: Supplementary file 9 — Supplementary file9 (PDF 426 kb) [file 464_2021_8906_MOESM9_ESM.pdf]

Reinier de Graaf

Wetenschapsraad

Direct line: 015 260 41 01
Room number: G.3.013
E-mail: wetenschapsraad@rdgg.nl

Alexander L.A. Bloemendaal
Department of Surgery
Reinier de Graaf Gasthuis
Delft, The Netherlands

Date: November 1th 2021

de Gravin

Postal address
Postbus 5011
2600 GA Delft

Visiting address
Reinier de Graafweg 9
2625 AD Delft
015 260 30 60

www.reinierdegraaf.nl

Concern: Study 'Validation of the Portable Virtual Reality Training System for Robotic Surgery (PoLaRS)'

Dear doctor Bloemendaal,

You have submitted the study protocol 'Validation of the Portable Virtual Reality Training System for Robotic Surgery (PoLaRS)' to the scientific board of the Reinier de Graaf Hospital, whether this study is subject to the Medical Research Involving Human Subjects Act (WMO) by the IRB.

The broad definition for medical research given by the WMO is: 'Medical/scientific research is research which is carried out with the aim of finding answers to a question in the field of illness and health (etiology, pathogenesis, signs/symptoms, diagnosis, prevention, outcome or treatment of illness), by systematically collecting and analysing data'.
The goal of the study under review is to gain insights in differences in the learning curve of medical students, in robotics handling, with the use of a training program. This pilot study therefore does not involve or affect patient care, as defined by the WMO.
Secondly, the study subjects are not submitted to a potentially hazardous environment. That is, the working area only contains non-actuated (passive) components and the motion sensors work at low harmless voltages.
Our conclusion is that the submitted study is out of the scope of the Human Subjects Act (WMO) and does not need a formal submission to an accredited MREC.

We wish you lots of success with the proceedings of the submission of the article.

With best regards,
On behalf of the Scientific board

Dr. Frans van der Horst
Chair of Scientific board Reinier de Graaf Gasthuis
